# The role of bone grafts in displaced intra-articular calcaneal fractures: A prospective study protocol

**DOI:** 10.1097/MD.0000000000023740

**Published:** 2020-12-24

**Authors:** Zhi-xiang He, Zheng-hao Lu, Jun Ou, Zhi-liang Wu

**Affiliations:** a Department of Hand Surgery; b Department of Spine Surgery, Affifiliated Nanhua Hospital of University of South China, Hengyang, Hunan, China.

**Keywords:** bone graft, displaced intra-articular calcaneal fractures, randomized controlled trial, study protocol

## Abstract

**Background:**

Whether the bone graft is needed in treating the displaced intra-articular calcaneal fractures (DIACFs) is still controversial. Therefore, in our study, we will explore the results of 2 approaches for the DIACFs surgical treatment.

**Methods:**

The present report follows the Consolidated Standards of Reporting Trials (CONSORT) guidelines. All patients will be assigned randomly into 2 different groups through tossing the coins. Three experienced surgeons are assigned randomly to each group to implement the surgeries utilizing any of the surgical approach. Assignments are concealed in a sealed opaque envelope. Patients who meet the following conditions will be included in this experiment:

(1) DIACFs (greater than 2 mm) involve Sanders Type IIC, Type IIB as well as some Type III; and
(2) surgical treatment can be implemented within 7 days after injury. Some patients with obvious swelling can wait for 2 weeks before operation;
(3) patients with closed fracture and;
(4) unilateral fracture. In the 2 groups, the patients will follow the standard postoperative protocols.

Patients are asked to finish 2 questionnaires, namely, American Orthopaedic Foot and Ankle Society score and short form 36. The ranges of motion of the ankle and the subtalar joint will be also measured. Postoperative complications such as deep infection, wound infection, and wound edge necrosis, the injury of sural nerve, and hematoma are recorded.

**Results:**

Our study can provide significant information on the necessity of bone graft in DIACFs internal fixation treatment.

**Trial registration:**

This study protocol was registered in Research Registry (researchregistry6246).

## 1 Introduction

The calcaneal fractures account for approximately 1% to 2% of all the fractures in human body, with 11.5 cases per 100,000 people occur every year, of which more than half are displaced intra-articular calcaneal fractures (DIACFs).^[1–4]^ Owing to the constant pain, stiffness, and abnormal gait, DIACFs usually lead to permanent disability and reduce the quality of life, which predominantly occur mainly in active young patients. As a result, these fractures can lead to severe long-term disability and high socioeconomic impact.^[5–7]^

There is still controversy about whether DIACFs should be treated surgically or non-surgically. Historically, DIACFs have not been treated surgically because of unexpected surgical reduction and fixation.^[8]^ At present, the open reduction and internal fixation (ORIF) is regarded as a reference standard in treating the DIACFs, as anatomical reduction is important for a good functional outcome of the foot and ankle.^[9–11]^

Nevertheless, whether the bone graft is needed in treating the DIACFs is still controversial. Orthopedics who insist on bone grafts believe that it will promote fracture healing, prevent the arthritis after traumatic, result in complete weight bearing early, and enhance the mechanical strength, and helping to prevent severe collapse in the late stage.^[12,13]^ Those who do not like bone grafts suggest that internal fixation can support the surface of the joint adequately, while extra bone grafting can enhance the pain after operation and the blood loss, increase the complications, infection rate, and incidence rate of donor site.^[13,14–17]^

In recent years, a number of clinical trials have assessed the influence of non-bone grafts and bone grafts in the DIACFs surgical treatment. However, the outcomes of these studies are inconsistent, and the number of high-quality prospective trials conducted is limited, thus it is impossible to determine whether bone grafting is necessary for surgical treatment of intra-articular calcaneal fractures.^[18,19]^ Therefore, in our study, we will explore the results of 2 approaches for the DIACFs surgical treatment (ORIF with or without bone grafts). Our study can provide significant information on the necessity of bone graft in DIACFs internal fixation treatment.

## 2 Materials and methods

### 2.1 Patients and design

This trial has been approved by the institutional review committee of our hospital (Affifiliated Nanhua Hospital of University of South China, protocol number 172003) and has been prospectively registered on Research Registry (researchregistry6246). The present report follows the Consolidated Standards of Reporting Trials (CONSORT) guidelines. All patients will be assigned randomly into 2 different groups through tossing the coins. Three experienced surgeons are assigned randomly to each group to implement the surgeries utilizing any of the surgical approach. Assignments are concealed in a sealed opaque envelope.

Patients who meet the following conditions will be included in this experiment:

(1) DIACFs (greater than 2 mm) involve Sanders Type IIC, Type IIB as well as some Type III; and
(2) surgical treatment can be implemented within 7 days after injury. Some patients with obvious swelling can wait for 2 weeks before operation;
(3) patients with closed fracture and;
(4) unilateral fracture.

While patients with the following conditions will be excluded:

(1) patients with known systemic or local infection;
(2) patients with the medical contraindications (involving diabetes and severe nerve or vascular injury);
(3) open fractures, Sanders Type IIA and Sanders Type IV. Sanders Type IV is excluded due to the posterior facet is too comminuted to allow rapid reduction. And Sanders Type IIA is excluded owing to we believe that the displaced posterior articular surface is too small for reduction and fixation.

### 2.2 Techniques and interventions

The patient is placed on the luminous operating table in lateral decubitus position. And the upper thigh is fixed with tourniquet, and an incision with L shape is made on lateral side. Through a sharp dissection, the subperiosteal flap is formed to protect the sural nerve and surrounding soft tissue. After the fracture line is clearly visible, the broken fragments of lateral wall of fracture are lifted to serve as a covering for the calcaneus. This clearly exhibits the collapsed posterior articular surface. Through utilizing periosteal stripping as the lever or placing the osteotome on the lower side, the collapsed bone mass is lifted to its normal upper position. Subsequently, the temporary fixation of posterior facet is carried out from thalamic section to sustentaculum through Kirchnerwires. The valgus/varus alignment and reduction of calcaneocuboid joint, posterior articular surface, calcaneal ulnar joints, susten-taculum, and anterior process are examined by fluoroscopy with Harris axial, lateral, as well as serial Broden images. At this stage, the bone defects are found as posterior facet is lifted from the impact site, and in group A, the cancellous allograft is tightly packed into patients’ calcaneus bone in accordance with the surgeon's preference. No bone graft is performed in the group B, which reached the height of calcaneus.

### 2.3 Rehabilitation

In the 2 groups, the patients will follow the standard postoperative protocols. On the third day after operation, the patients begin to walk without weight-bearing. When the patient can tolerate, the mobilization trainings of subtalar and ankle joint will be begun. After 6 weeks, patients begin to conduct gradual weight-bearing, starting at 25 percent of their weight. Complete weight bearing is allowed after twelve weeks, provided that the stabilized and reduced fracture position is still unchanged, and that there are clinical radiological signs of the bone healing at the time (no tenderness, swelling or pain at the site of the clinical fracture, and no fracture line is visible on the X-rays).

### 2.4 Outcomes

Both subjective and objective outcome-scoring systems will be assessed in our study. The CT scan and radiographs are utilized to evaluate the degree of fracture reduction and whether to maintain fracture reduction. Patients are asked to finish 2 questionnaires, namely, American Orthopaedic Foot and Ankle Society score and short form 36. short form 36 is a recognized result module of patient and AFASS is a result evaluation by a tested physician. The ranges of motion of the ankle and the subtalar joint will be also measured. Postoperative complications such as deep infection, wound infection, and wound edge necrosis, the injury of sural nerve, and hematoma are recorded.

### 2.5 Statistical analysis

The statistical analysis can be carried out through applying the software of SPSS v. 24 (IBM Corp., Armonk, NY) for Windows. The continuous data with normal distribution can be represented as mean ± SD. The non-paired t test and Mann-Whitney U test are respectively utilized for the comparison of continuous variables with near normal distribution and non-normal distributions. The classification data are analyzed statistically with Fisher exact test or Chi-square test. *P* value less than .05 suggests that there is statistical significance.

## 3 Discussion

The treatment of displaced intra-articular calcaneal fractures is still controversial. Nonsurgical treatment may be recommended for patients with minor intra-articular displacement and patients with surgical contraindications. Buckley et al believed that ORIF have better functional effects on the treatment of displaced intra-articular calcaneal fractures than non-surgical treatment. At present, ORIF is regarded as a reference standard in treating the DIACFs, as anatomical reduction is important for a good functional outcome of the foot and ankle. Whether bone graft is needed in treating the DIACFs is still controversial. In our study, we will explore the results of 2 approaches for the DIACFs surgical treatment (ORIF with or without bone grafts). Our experiment can provide significant information on the necessity of bone graft in DIACFs internal fixation treatment.

## Author contributions

**Conceptualization**: Zhi-liang Wu.

**Data curation**: Zhi-xiang He, Zheng-hao Lu.

**Formal analysis**: Zhi-xiang He, Zheng-hao Lu.

**Funding acquisition**: Zhi-liang Wu.

**Investigation**: Zhi-xiang He, Zheng-hao Lu.

**Methodology**: Jun Ou.

**Resources**: Zhi-liang Wu.

**Software**: Jun Ou.

**Supervision**: Zhi-liang Wu.

**Validation**: Jun Ou.

**Visualization**: Jun Ou.

**Writing – original draft**: Zhi-xiang He.

**Writing – review & editing**: Zhi-liang Wu.

## References

[1] Saß M Rotter R Mittlmeier T Minimally invasive internal fixation of calcaneal fractures or subtalar joint arthrodesis using the Calcanail. Oper Orthop Traumatol 2019;31:149–64. 30413845 10.1007/s00064-018-0576-2

[2] Buckley R Tough S McCormack R Operative compared with nonoperative treatment of displaced intra-articular calcaneal fractures: a prospective, randomized, controlled multicenter trial. J Bone Joint Surg Am 2002;84:1733–44. 12377902 10.2106/00004623-200210000-00001

[3] Mehta CR An VVG Phan K Extensile lateral versus sinus tarsi approach for displaced, intra-articular calcaneal fractures: a meta-analysis. J Orthop Surg Res 2018;13:243. 30249288 10.1186/s13018-018-0943-6 PMC6154938

[4] Zhao W Zhang Y Comparison and predictive factors analysis for efficacy and safety of Kirschner wire, anatomical plate fixation and cannulated screw in treating patients with open calcaneal fractures. Medicine (Baltimore) 2019;98:e17498. 31651853 10.1097/MD.0000000000017498 PMC6824657

[5] Zhang Z Wang Z Zhang Y Risk factors for increased postoperative drainage of calcaneal fractures after open reduction and internal fixation: an observational study. Medicine (Baltimore) 2018;97:e11818. 30095652 10.1097/MD.0000000000011818 PMC6133466

[6] Correoso Castellanos S García Galvez A Lajara Marco F Intra-articular calcaneal fractures. Do locking plates keep the reduction better than conventional plates? Rev Esp Cir Ortop Traumatol 2019;63:383–8. 31451428 10.1016/j.recot.2019.05.003

[7] Griffin D Parsons N Shaw E Operative versus non-operative treatment for closed, displaced, intra-articular fractures of the calcaneus: randomised controlled trial. BMJ 2014;349:g4483. 25059747 10.1136/bmj.g4483 PMC4109620

[8] Marouby S Cellier N Mares O Percutaneous arthroscopic calcaneal osteosynthesis for displaced intra-articular calcaneal fractures: systematic review and surgical technique. Foot Ankle Surg 2020;26:503–8. 31320206 10.1016/j.fas.2019.07.002

[9] Nosewicz TL Dingemans SA Backes M A systematic review and meta-analysis of the sinus tarsi and extended lateral approach in the operative treatment of displaced intra-articular calcaneal fractures. Foot Ankle Surg 2019;25:580–8. 30321924 10.1016/j.fas.2018.08.006

[10] Liu GT Vanpelt MD Lalli T Surgical management of displaced intra-articular calcaneal fractures: what matters most? Clin Podiatr Med Surg 2019;36:173–84. 30784529 10.1016/j.cpm.2018.10.002

[11] Berberian W Sood A Karanfilian B Displacement of the sustentacular fragment in intra-articular calcaneal fractures. J Bone Joint Surg Am 2013;95:995–1000. 23780537 10.2106/JBJS.L.01498

[12] Cao H Li YG An Q Short-term outcomes of open reduction and internal fixation for sanders type III calcaneal fractures with and without bone grafts. J Foot Ankle Surg 2018;57:7–14. 29037927 10.1053/j.jfas.2017.05.037

[13] Chen L Zhang G Hong J Comparison of percutaneous screw fixation and calcium sulfate cement grafting versus open treatment of displaced intra-articular calcaneal fractures. Foot Ankle Int 2011;32:979–85. 22232815 10.3113/FAI.2011.0979

[14] Duymus TM Mutlu S Mutlu H Need for bone grafts in the surgical treatment of displaced intra-articular calcaneal fractures. J Foot Ankle Surg 2017;56:54–8. 27839662 10.1053/j.jfas.2016.08.004

[15] Singh AK Vinay K Surgical treatment of displaced intra-articular calcaneal fractures: is bone grafting necessary? J Orthop Traumatol 2013;14:299–305. 23670493 10.1007/s10195-013-0246-y PMC3828503

[16] Feng Y Shui X Wang J Comparison of percutaneous cannulated screw fixation and calcium sulfate cement grafting versus minimally invasive sinus tarsi approach and plate fixation for displaced intra-articular calcaneal fractures: a prospective randomized controlled trial. BMC Musculoskelet Disord 2016;17:288. 27422705 10.1186/s12891-016-1122-8 PMC4946135

[17] Gusic N Fedel I Darabos N Operative treatment of intraarticular calcaneal fractures: Anatomical and functional outcome of three different operative techniques. Injury 2015;46:S130–3. 26606987 10.1016/j.injury.2015.10.061

[18] Longino D Buckley RE Bone graft in the operative treatment of displaced intraarticular calcaneal fractures: is it helpful? J Orthop Trauma 2001;15:280–6. 11371794 10.1097/00005131-200105000-00008

[19] Brand A Klöpfer-Krämer I Böttger M Gait characteristics and functional outcomes during early follow-up are comparable in patients with calcaneal fractures treated by either the sinus tarsi or the extended lateral approach. Gait Posture 2019;70:190–5. 30884444 10.1016/j.gaitpost.2019.03.007

